# Biodegradation of high molecular weight hydrocarbons under saline condition by halotolerant *Bacillus* *subtilis* and its mixed cultures with *Pseudomonas* species

**DOI:** 10.1038/s41598-022-17001-9

**Published:** 2022-08-02

**Authors:** Hassan Ghorbannezhad, Hamid Moghimi, Seyed Mohammad Mehdi Dastgheib

**Affiliations:** 1 Department of Microbial Biotechnology, School of Biology, College of Science, University of Tehran, Tehran, 1417864411 Iran; 2 Microbiology and Biotechnology Research Group, Research Institute of Petroleum Industry, Tehran, Iran

**Keywords:** Environmental biotechnology, Applied microbiology, Environmental microbiology, Environmental sciences

## Abstract

Biodegradation of high-molecular-weight petroleum hydrocarbons in saline conditions appears to be complicated and requires further investigation. This study used heavy crude oil to enrich petroleum-degrading bacteria from oil-contaminated saline soils. Strain HG 01, with 100% sequence similarity to *Bacillus* *subtilis*, grew at a wide range of salinities and degraded 55.5 and 77.2% of 500 mg/l pyrene and 500 mg/l tetracosane, respectively, at 5% w/v NaCl. Additionally, a mixed-culture of HG 01 with *Pseudomonas* *putida* and *Pseudomonas* *aeruginosa*, named TMC, increased the yield of pyrene, and tetracosane degradation by about 20%. Replacing minimal medium with treated seawater (C/N/P adjusted to 100/10/1) enabled TMC to degrade more than 99% of pyrene and tetracosane, but TMC had lesser degradation in untreated seawater than in minimal medium. Also, the degradation kinetics of pyrene and tetracosane were fitted to a first-order model. Compared to *B.* *subtilis*, TMC increased pyrene and tetracosane's removal rate constant (K_1_) from 0.063 and 0.110 per day to 0.123 and 0.246 per day. TMC also increased the maximum specific growth rate of *B.* *subtilis*, *P.* *putida*, and *P.* *aeruginosa*, respectively, 45% higher in pyrene, 24.5% in tetracosane, and 123.4% and 95.4% higher in pyrene and tetracosane.

## Introduction

Recalcitrant petroleum hydrocarbons have become one of the major causes of extensive damage to ecosystems^1,2^. Polycyclic aromatic hydrocarbons (PAHs) and long-chain aliphatics are hard to degrade, and some (mostly PAHs) display various toxicities like hemotoxic, carcinogenic, and teratogenic effects^3^. High-molecular-weight (HMW) PAHs and long-chain aliphatics are generally hard to degrade due to their complex structure, hydrophobicity, and low bioavailability^4,5^. Most of the low-molecular-weight (LMW) hydrocarbons have been removed in many areas with long-term petroleum contamination. Conversely, many persistent pollutants such as HMW PAHs and long-chain aliphatics remain^1,6^. For example, while crude oil has a half-life of 7–14 days in seawater, some HMW PAHs and long-chain aliphatics, such as pyrene, chrysene, tetracosane, and hexacosane, have half-lives up to 151, 182, 82, and 96 days, respectively^7,8^.

Bioremediation (converting organic contaminants into harmless, simple chemicals by biological processes) can be accomplished through natural attenuation, bioaugmentation, and biostimulation^9^. Regardless of the method, successful bioremediation requires using specialized organisms capable of degrading pollutants^10^. Natural attenuation has a poor remedial outcome in many cases, and there is a risk of residual contamination after a biostimulation operation^11^. For degrading recalcitrant hydrocarbons, employing bioaugmentation processes by adding fast-growing and easily culturable microorganisms with high stability, stress tolerance, and enduring high concentrations of pollutants could be beneficial^11,12^. Previous research showed that bioaugmentation of oil-degrading bacteria increased total petroleum hydrocarbons (TPH) and PAHs removal to 52 and 87%, respectively, from 47 and 59% by biostimulation and 37 and 42% by natural attenuation^13^.

Petroleum-contaminated environments contain various hydrocarbons, making detoxification incredibly hard for an individual organism^4^. Using consortiums, mixed-cultures, and even adding various physiochemistry agents are different approaches for enhancing bioremediation efficiency^14,15^. In mixed-cultures, in addition to increasing the range of degradable substrates, by-products and intermediates created by one member can be consumed by others, or another organism can improve the degradation efficiency of others by producing biosurfactants^16,17^. Among oil-degrading microorganisms, bacteria are the most active and crucial group mainly because of their widespread distribution, ease of handling, and abundance of metabolic pathways^18,19^. *Bacillus* and *Pseudomonas* species are two of the most efficient bioremediators. They have high growth rates, produce biosurfactants, and are adapted to a wide range of physicochemical conditions, making them ideal for clean-up processes^19–21^. Consequently, a mixed-culture of these bacteria could be suitable for degrading petroleum hydrocarbons, especially HMW hydrocarbons.

Considering that many of the world’s oil reservoirs are originated from saline environments, petroleum contamination is likely to be associated with a wide range of salinity in the proximity of evaporites and aqueous environments^22^. Additionally, one of the characteristics shared by many petrochemical and petroleum refining industries is the high salinity of their enormous wastewater^23^. Information on remediation by halophilic or halotolerant microorganisms is scarce, and various issues have stayed unsolved^24^. Due to the lower microbial diversity and the reduced solubility of hydrocarbons and oxygen, biodegradation of hydrocarbons is decreased at higher salinities^25^. In such cases, operating the bioaugmentation process of contaminated saline environments with halophilic or halotolerant microorganisms is essential and efficient, as was shown previously by several research groups^24,26,27^.

Pyrene, a 4-ring PAH, and tetracosane, a 24-carbon aliphatic, have often been used as model hydrocarbons due to their abundance and structural similarity with other HMW hydrocarbons^4,28^. This study tested the degradation efficiency of pyrene and tetracosane by a newly isolated *B.* *subtilis* at various salinities (0–10% w/v). Then, due to the HMW of pyrene and tetracosane and the complicity of their degradation for a single microorganism, the effects of different mixed-cultures with two *Pseudomonas* species were investigated. In pure and mixed-cultures, the degradation efficiency, activity, and growth patterns of *B.* *subtilis*, *P.* *aeruginosa* (a known biosurfactant producer), and *P.* *putida* (a known aromatic degrader) were investigated. Additionally, the effects of using common salt (NaCl) versus seawater on the degradation process were evaluated.

## Results and discussion

The results of the isolation process and selection of the best strain based on the criteria of degrading heavy crude oil (1% w/v) are presented in Supplementary File 1. Figure 1 of Supplementary File 1 shows that the HG 01 strain with 59.5% degradation had the best result and was chosen for further studies. The molecular identification and sequence comparison results of 16s rDNA illustrated 100% similarity to *Bacillus* *subtilis* (Supplementary File 1). The 16s rRNA sequence of the *Bacillus* *subtilis* HG 01 was deposited to the genetic sequence database (GenBank) at NCBI with accession number MW548284.

Based on the salinity assay’s results, the *B.* *subtilis* HG 01 growth curve showed an acceptable tolerance against salinity up to 20% w/v (Fig. 2 in Supplementary File 1). Figure 2 in Supplementary File 1 shows the better growth of HG 01 in 5% NaCl. The curve of *B.* *subtilis* HG 01 growth had an acceptable tolerance against salinity up to 20% w/v and a good growth up to 10% w/v.

The results of the oil spreading test (Table 1 in Supplementary File 1) show the ability of *B.* *subtilis* HG 01 in producing biosurfactants on all three carbon sources. The oil spreading activity of *B.* *subtilis* HG 01 supernatants from the pyrene cultures was less than half and one-third as effective as the supernatants from glucose and tetracosane. The supernatants of *P.* *aeruginosa* ATCC 10145 (a well-known biosurfactant producer) had a significant oil spreading activity on all three carbons sources. However, its activity decreased by about 10% and 32% when the carbon source changed from glucose to tetracosane and pyrene. The supernatant of *P.* *putida* ATCC 12633 had no statistically significant oil spreading activity.

### Degradation of HMW hydrocarbons by *B. subtilis* HG 01

**Figure 1:**
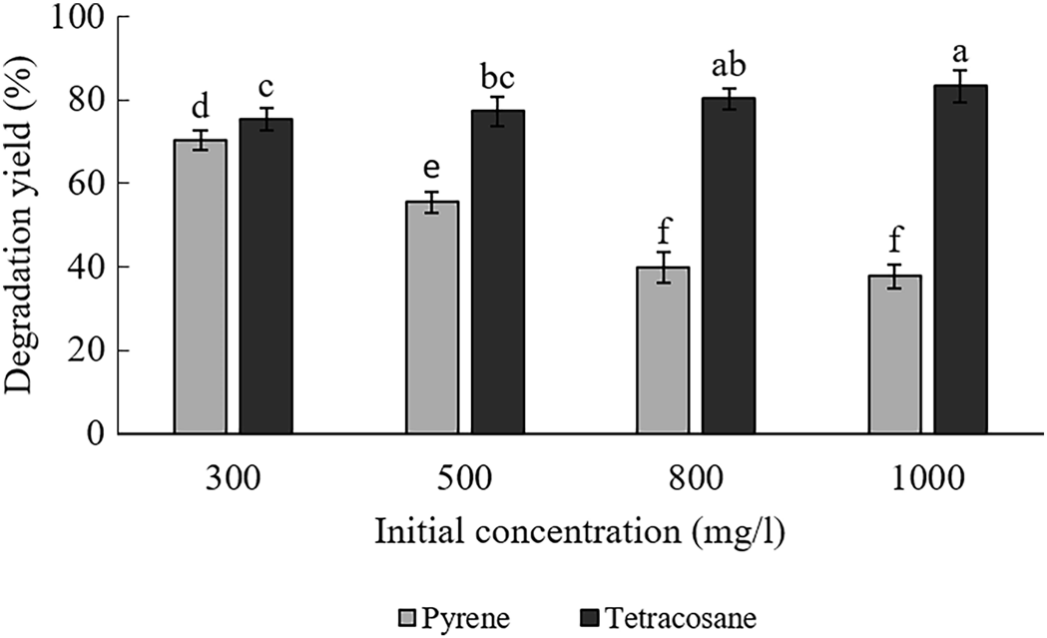
Degradation yield of pyrene and tetracosane by *B.* *subtilis* HG 01 at different concentration. Data presented as mean ± S.D. (n = 3). Different alphabets between columns represent significance at p < 0.05 after applying post hoc Tukey’s test.

A degradation test on pyrene and tetracosane at different concentrations (300, 500, 800, and 1000 mg/l) was performed to evaluate the potential of *B.* *subtilis* HG 01 in removing HMW hydrocarbons in 5% w/v NaCl. The experimental data of the degradation test are provided in Fig. 1. The yield of pyrene degradation falls from 70.2 to 37.7%, while its concentration rises from 300 to 1000 mg/l. The findings support some prior research that links pyrene’s degradation yield with its initial concentration^3,26^. For example, when the initial pyrene concentration was increased from 300 to 1000 mg/l, the degradation yield of pyrene by *Basidioascus* *persicus* EBL-C16 was reduced by about 45%^26^. Another study indicated that increasing the initial concentration of pyrene from 10 to 600 mg/l resulted in a 60% reduction in pyrene degradation by *Acinetobacter* *baumannii* BJ5^3^. Similarly, in the current investigation, the yield of pyrene degradation decreased by around 32.5% as the concentration increased from 300 to 1000 mg/l.

Inversely, the degradation yield of tetracosane gradually increased with a gentle slope along with concentration and reached 83.4% at 1000 mg/l as against 75.3% at 300 mg/l. There are similarities between the activities expressed by *B.* *subtilis* HG 01 toward tetracosane degradation and those described by another study. In that study, the effect of tetracosane initial concentration (from 500 to 1500 mg/l) on the growth of thermophilic *B.* *subtilis* YB7 was investigated. It was shown that by increasing the density from 500 to 1000 mg/l, the CFU count improved about 18% and dropped by almost 40% until 1500 mg/l. This thermophilic *Bacillus* strain degraded about 64, 91, and 89% of 1000 mg/l tetracosane, respectively, at 35, 50, and 75 °C in 10 days^29^.

The differences between pyrene and tetracosane degradation changes can be explained differently. A possible explanation is the lesser potency of *B.* *subtilis* HG 01 in producing biosurfactant on pyrene. So, even with increasing concentration, the mass of bioavailable pyrene does not change much. Another possible explanation might be the higher toxicity of pyrene; so, as the concentration increases, bacterium cells are in contact with more toxic effects^30^. Moreover, the PAH degradation process is more complicated than aliphatics, requires more enzymes, and takes extra steps. Hence, most microorganisms prefer alkane substrates mainly due to lesser adaptation time and enzymes’ contribution to the degradation of aliphatics^1,31^.

In general, many bacteria can degrade long-chain aliphatics or PAHs, but fewer strains can degrade them simultaneously^22,32^. Like HG 01, other bacteria such as *P.* *aeruginosa* DQ8 and *Staphylococcus* sp. CO100 were able to degrade long-chain aliphatics and HMW PAHs as well. *P.* *aeruginosa* DQ8 degraded 80% of triacontane (200 mg/l) and 50% of tetrocontane (1000 mg/l) in 7 days and 40.5% of pyrene in 12 days from a mixture of four aromatic hydrocarbons (each at 40 mg/l)^30^. Besides, *Staphylococcus* sp. CO100 was able to degrade up to 72% of aliphatics in 1% (v/v) crude oil and grow on phenanthrene, fluoranthene and pyrene (at 100 mg/l) up to 3% w/v NaCl^33^. Due to the substrate’s initial concentrations, HG 01 removed pyrene up to 70.2% (at 300 mg/l) and tetracosane up to 86.4% (at 1000 mg/l) at 5% w/v salinity, indicating the high potency of this strain in bioremediation of saline petroleum-contaminated aquatics.

### Synergistic effect of *Pseudomonas* species mixed with *B. subtilis* HG 01 in degradation of pyrene and tetracosane by *B. subtilis* HG 01

A considerable amount of literature has displayed the advantages of using mixed-cultures, consortiums, or even co-cultures of two microorganisms over pure cultures^26,34^. So, to evaluate the succession of this possibility, a PAH degrader *P.* *putida* ATCC 12633 and a biosurfactant producer *P.* *aeruginosa* ATCC 10145 (in equal quantities) were used to form binary/ternary mixed-cultures with *B.* *subtilis* HG 01. The binary mixed-culture of *B.* *subtilis* with *P.* *aeruginosa* and *P.* *putida* were named BMC 1 and BMC 2, respectively; and the ternary mixed-culture was named TMC.

**Figure 2:**
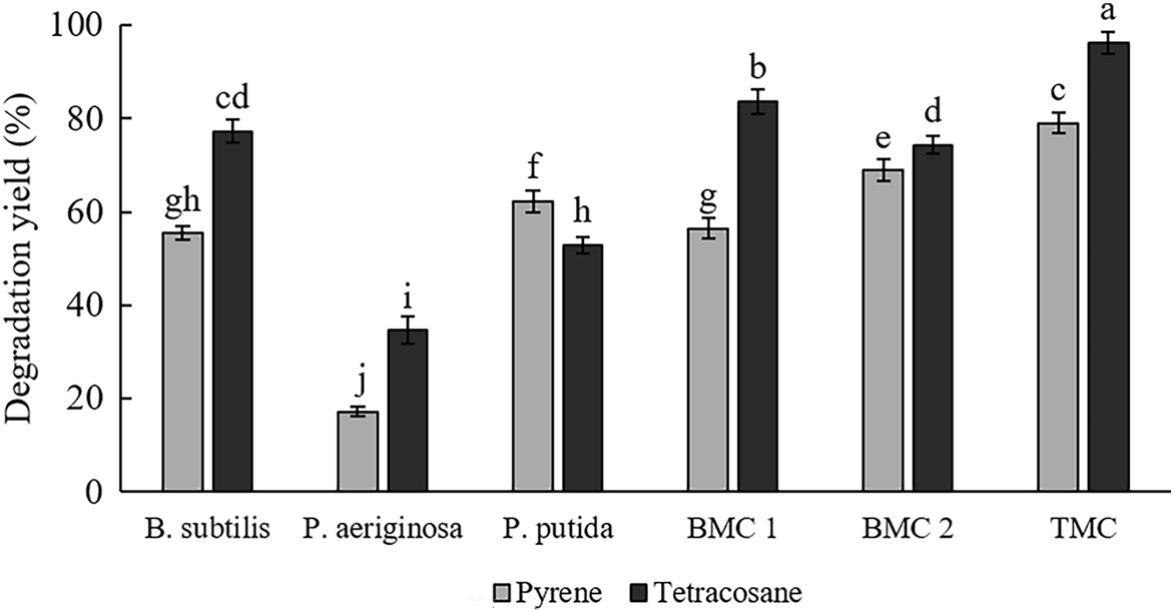
Effect of using *Pseudomonas* species mixed-culture with *B.* *subtilis* HG 01 on pyrene and tetracosane degradation. Each value is shown as mean ± S.D. (n = 3). Different letters indicate significant differences (p < 0.05).

The obtained results showed that using *P.* *putida* ATCC 12633 had more effect on pyrene degradation than tetracosane and increased the degradation yield of pyrene from 50.5 to 70% (Fig. 2). As mentioned before, *P.* *putida* ATCC 12633 is a PAH degrader and degrades pyrene through the phthalic acid pathway^35^, the same as *Bacillus* species^36^. Therefore, the synergistic effects of similar enzymes in the metabolic pathways could be attributed to the increase of pyrene degradation. In collaboration, another study found an 11.4% increase in pyrene degradation by employing a co-culture composed of *P.* *putida* ATCC 12633 and *Basidioascus* *persicus* EBL-C16 in a 21-day incubation, which both used phthalic acid pathway for degradation of PAHs^26^. The increase in pyrene degradation with the co-culture of *P.* *putida* and *B.* *subtilis* was 19.5% in 14 days which is more than what was reported by the other study in less incubation period. The more genetic proximity of *P.* *putida* toward *B.* *subtilis,* compared to a fungus such as *B.* *persicus*, could be the reason for the mentioned difference.

On the contrary, *P.* *aeruginosa* ATCC 10145 had more positive effects on tetracosane degradation and increased it from 71.3 to 83.7%. *P.* *aeruginosa* ATCC 10145 is a biosurfactant producer, so its contribution to the performance of mixed-culture is increasing the bioavailability of carbon sources, and some prior studies have noted that generally, surface-active compounds have a lesser effect on PAHs^28,37^. In a similar study, the effect of Rhamnolipid (same as *P.* *aeruginosa* ATCC 10145 biosurfactant) produced by *Pseudomonas* sp. 0–2–2 at 0.1 g/l on the degradation of different aliphatics, PAHs, and biomarkers were investigated. The results showed that rhamnolipid even reduced the degradation yield of short-chain aliphatics and simple PAHs. However, rhamnolipid positively affected long-chain alkanes, HMW PAHs, and complex biomarkers degradation in the order of complex biomarkers > long-chain aliphatics > HMW PAHs^37^. Also, another study showed that both rhamnolipids and tween 80 performed better at increasing the degradation of tetracosane than pyrene^28^.

Finally, TMC had the best efficiency in degrading pyrene and tetracosane with 79.03 and 96.2%, respectively. In the TMC, three bacteria with relatively different roles are involved. *B.* *subtilis* is more effective in the degradation of tetracosane, and *P.* *putida* is more successful in degrading pyrene. Moreover, *P.* *aeruginosa*’s role is to increase the bioavailability of carbon sources through biosurfactant production. The consequence of this cooperation was a 28.6 and 24.9% increase in the extent of pyrene and tetracosane degradation. The present findings seem consistent with other research, which investigated the different roles of 5 culturable bacteria in a consortium. In the mentioned consortium, just the two *Mycobacterium* spp. had a complete gene set for pyrene degradation. *Novosphingobium* *pentaromativorans* and *Ochrobactrum* sp. degraded pyrene via different pathways. Finally, *Bacillus* sp. was only involved in enhancing the pyrene bioavailability by producing biosurfactants. The consortium showed threefold higher pyrene degradation than individual *Mycobacterium* species^4^. Furthermore, in a study investigating pyrene and tetracosane removal by different fungal, bacterial, and fungal-bacterial mixed-cultures, the bacterial mixed-culture had the lowest capacity^28^. Most of the species in this bacterial consortium had similar roles and behaviors in pyrene and tetracosane degradation. In comparison, the fungal-bacterial consortium had more diversity and success. These results indicate the importance of using different microorganisms with diverse capabilities and roles in removing recalcitrant hydrocarbons.

Many studies support the idea of employing mixed-cultures or consortiums for a better degradation process. Various research investigated the role of mixed-cultures in the degradation of crude oil and petroleum hydrocarbons^6,9,21,38^. Two investigations found that using a fungal-bacterial mixed culture increased crude oil (at 1% w/v) degradation between 12 and 15%^6,9^. Another one showed a 28% increase in pyrene degradation (at 75 mg/l) after applying a fungal-bacterial mixed-culture^21^. Even a bacterial-micro algal mixed-culture consisting of *Scenedesmus* *obliquus*, *Sphingomonas* sp., and *Burkholderia* *cepacia* increased the degradation yield of a mixture of n-alkanes by about 20%^38^.

### Comparison of pyrene and tetracosane degradation in seawater and mineral medium with different salinities

However, many investigations have simulated the efficacy of the bioremediation process on petroleum contaminated soils through microcosm or mesocosm^39,40^; few studies have dealt with such simulations in seawater^41^. The ability of *B.* *subtilis* HG 01 and TMC in removing petroleum pollutants from an untreated and treated seawater (adding nitrogen and phosphorous to reach a C/N/P ratio of 100/10/1) was evaluated and compared to mineral medium Bushnell-Haas (BH) with 0, 2.5, 5, and 10% w/v salinity. With the entry of hydrocarbon pollutants into the seawater, the C/N/P ratio becomes unbalanced, and therefore microorganisms cannot perform to the best of their ability. The minimum C/N/P ratio required for the proper function of microorganisms is 100/10/1^42^. This ratio is even slightly higher in the BH medium; So, a seawater sample was treated to eliminate the effects of lacking nitrogen and phosphorus sources from the results.

The results obtained from the analysis are compared in Table 1. As can be seen, there are no significant differences in pyrene and tetracosane degradation between 0 and 2.5% w/v salinities, but with the increase of NaCl concentration to 5% w/v, pyrene and tetracosane degradation increased about 10 and 14% for *B.* *subtilis*, and 4 and 7% for TMC. Also, 10% w/v salinity had the lower pyrene and tetracosane degradation yield with 38.5 and 51.2% for *B.* *subtilis* and 40.4 and 49.6% for TMC. *B.* *subtilis* HG 01 showed a higher increase in degradation yield than TMC when the salinity was increased to 5% w/v. In addition, the degradation of the HG 01 strain and TMC were not significantly different in 10% w/v NaCl, which suggests that *B.* *subtilis* is more resistant to salt than *P.* *putida* and *P.* *aeruginosa*. However, the degradation of pyrene and tetracosane slightly decreased in untreated seawater (about 4–6%), but it showed a 14.28 and 17% increase by *B.* *subtilis* in treated seawater compared to BH with 5% w/v NaCl. Also, TMC reached the same amount of degradation in 10 days at treated seawater compared to 14 days at BH with 5% w/v NaCl (Table 1). In a similar observation, an 48% increase in pyrene degradation by yeast *Basidioascus* *persicus* EBL-C16 was reported after switching BH with 2.5% w/v NaCl to seawater^26^. The amount of different minerals in the Persian Gulf seawater (without supplementation) is presented in Supplementary File 2. Persian Gulf seawater has much more Magnesium (Mg) and Calcium (Ca) than BH. These two elements are required for cell growth, cell maintenance, initial attachments, and biofilm maturation through physiochemical interactions, gene regulation, and bio-macromolecular structural modification^43^. Also, organic carbon compounds formed from inorganic carbon photosynthesis in the structure of carbohydrates and amino acids are found in the sea’s surface waters. However, these organic carbon compounds have low concentrations but can be considered as a secondary carbon source that helps the growth of bacteria^44^.

**Table 1:** Degradation of pyrene and tetracosane by *B.* *subtilis* and TMC at different salinities. Values are mean (n = 3) + SD.

Cultures	Pyrene degradation (%)			Tetracosane degradation (%)		
	Day 10	Day 12	Day 14	Day 10	Day 12	Day 14
*B.* *subtilis* (0% NaCl)	31.9 ± 1.8	39.2 ± 1	44.8 ± 2.9	45.2 ± 2	57.4 ± 2.6	62.5 ± 1.8
TMC (0% NaCl)	58.3 ± 2.5	70.3 ± 2.1	74.8 ± 2.8	69.9 ± 2.6	75.9 ± 1.4	88.1 ± 2.1
*B.* *subtilis* (2.5% NaCl)	33 ± 2.9	41.2 ± 1.4	45 ± 1.9	48.4 ± 2.5	59.1 ± 2.5	63.6 ± 2.9
TMC (2.5% NaCl)	57.3 ± 2.6	65.1 ± 1.3	73.7 ± 2.5	70.5 ± 1.1	77.6 ± 1.6	89.4 ± 1.6
*B.* *subtilis* (5% NaCl)	38.6 ± 1.1	50 ± 1.8	55.5 ± 1.5	54.1 ± 1.6	71.4 ± 2.2	77.3 ± 1.9
TMC (5% NaCl)	67.4 ± 2	74.5 ± 2.1	79.3 ± 2.3	85 ± 2.2	94.3 ± 1.8	96.2 ± 2.3
*B.* *subtilis* (10% NaCl)	26.6 ± 2.3	31.5 ± 1.7	38.5 ± 1.8	39.7 ± 1.6	43.2 ± 2.7	51.2 ± 1.9
TMC (10% NaCl)	24.5 ± 1.4	30.7 ± 2.5	40.4 ± 2.2	37.1 ± 1.3	44.4 ± 2.6	49.6 ± 2.4
*B.* *subtilis* (TS)	51.2 ± 2.1	60.4 ± 1.7	66.7 ± 2.4	73.4 ± 3.1	81.2 ± 3.9	89.3 ± 3.3
TMC (TS)	82.1 ± 1.9	91.3 ± 2.7	> 99	94.4 ± 3.2	> 99	> 99
*B.* *subtilis* (UTS)	35.6 ± 2.9	46 ± 2.1	52.9 ± 1.2	53.1 ± 2.4	64.4 ± 1.8	73.3 ± 2.1
TMC (UTS)	61.8 ± 1.7	70.1 ± 1.4	74.9 ± 2.2	76.2 ± 2.9	81.3 ± 2.9	89.2 ± 1.9

A similar study reported up to 92.1 and 42.4% degradation yield, respectively, for medium and long-chain aliphatic hydrocarbons from untreated seawater supplemented with a 1000 mg/l mixture of aliphatic compounds by a co-culture of *B.* *methylotrophicus* SSNPLPB5 and *P.* *sihuiensis* SNPLPB7 in 46 days. Also, these two bacteria degraded 46% of anthracene, 33.9% of phenanthrene, and 35.3% of pyrene from a 1000 mg/l PAH mixture^10^. These comparisons show that balancing the C/N/P ratio accelerates the rate of degradation. Likewise, another study used a bacterial consortium to remediate a naturally polluted seawater with about 1000 mg/l petroleum pollution and indicated about 79% TPH loss in 14 days^41^. *B.* *subtilis* HG 01 showed an adequate performance in seawater with a higher degradation yield than *B.* *methylotrophicus* SSNPLPB5 and *P.* *sihuiensis* SNPLPB7. These results show that *B.* *subtilis* HG 01 and TMC can be considered promising means for remediation of naturally polluted seawater through bioaugmentation.

Finally, it should be noted that autochthonous microorganisms can degrade a more significant number of different pollutants by biological pathways than allochthonous microorganisms with higher rates and efficiencies. However, allochthonous microorganisms were used in this study, and the seawater was sterilized by autoclave before inoculation and contained no indigenous microorganisms. Even one research has shown that in the case of inoculation of allochthonous biomass in an environment with indigenous biomass, most of the removal is done by indigenous ones^45^. Even one study stated that autochthonous biomass could lower the COD of wastewater twice as much as allochthonous microorganisms (75% COD removal for autochthonous microorganisms and 40% COD removal for allochthonous Microorganism)^46^. Therefore, proper isolation and accumulation of microorganisms in a region, followed by re-inoculation of them into the environment, can yield superior outcomes.

### Growth rate and kinetics of pyrene and tetracosane degradation

**Figure 3:**
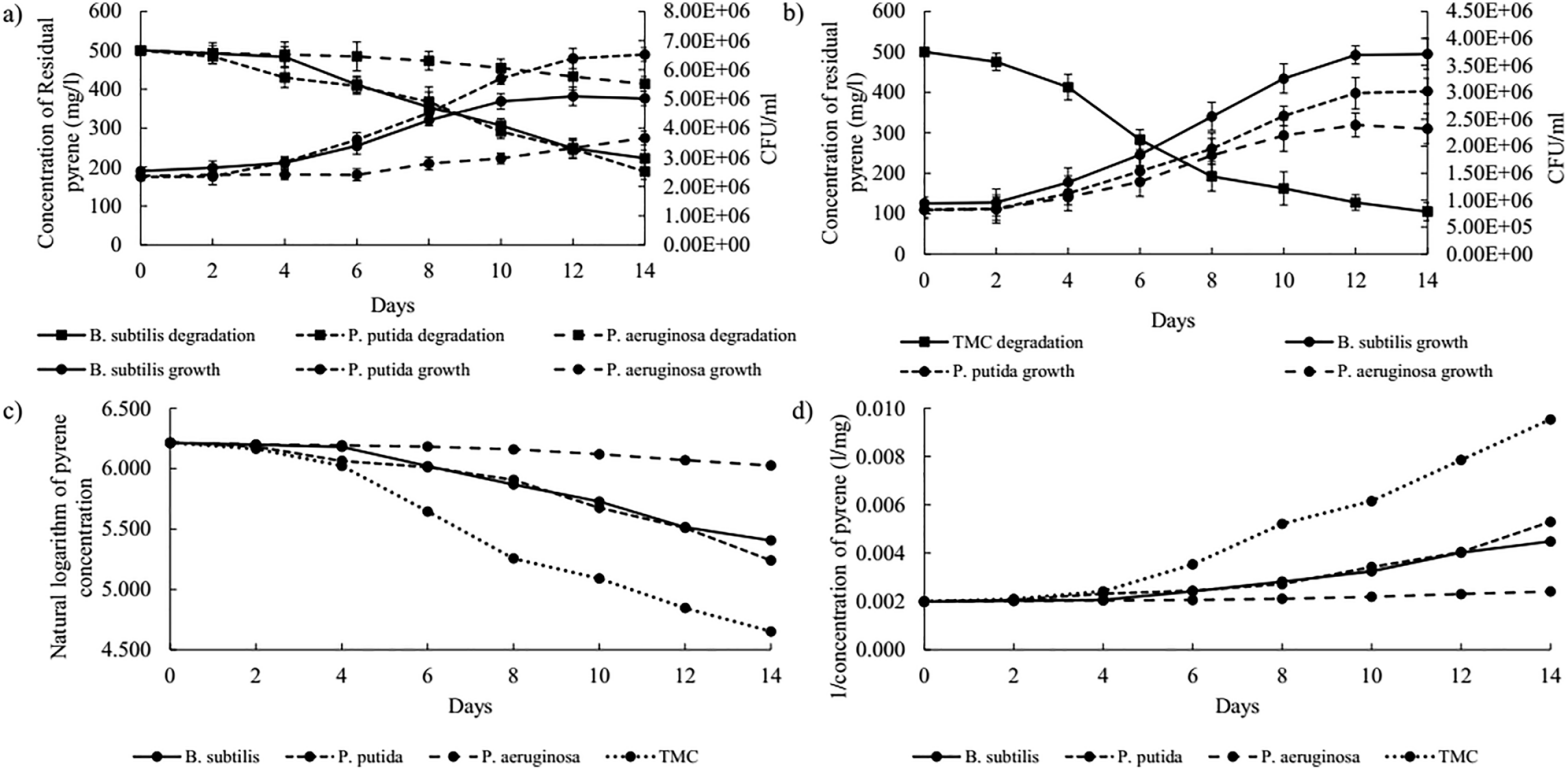
(**a**,**b**) Show the growth rate and degradation yield (zero order kinetic) of pyrene by pure cultures and TMC. Error bars along each point represent the standard deviation of the mean (n = 3). (**c**,**d**) Show the first order, and second order kinetic modeling of pyrene degradation by pure cultures and TMC.

Modeling of degradation kinetic is a requirement for predicting bioremediation processes and facilitating the control of biological processes; it is even an attractive approach to anticipate the changes in degradation activity and different microbial responses under the influence of specific environmental conditions^47^. To this end, a kinetic study on the degradation of pyrene and tetracosane by pure cultures and TMC was carried out. Pyrene degradation and biomass production diagrams over time are compared in Fig. 3a,b. This comparison showed three growth stages for pyrene degradation by *B.* *subtilis*, *P.* *putida*, and TMC. (1) at first, a lag phase occurred (four days for individual cultures and two days for TMC), in which *B.* *subtilis* did not degrade a significant amount of pyrene, (2) then, there was a logarithmic phase with the highest rate of degradation (6 days for individual cultures and 10 days for TMC), which *P.* *aeruginosa* growth greatly favored in TMC and (3) at the end, a stationary phase where the growth stopped, and degradation rate lowered but continued. Pyrene degradation still occurs when the bacteria are in the lag phase (for TMC) or stationary phase (*B.* *subtilis* and TMC). However, the removal rate is most significant when the bacteria are in the log phase. So, overall, these phases reveal that pyrene degradation is not confined to growth, but growth positively affects degradation. Besides, *P.* *aeruginosa* only had two phases: a 6-day lag phase without significant degradation followed by a slow exponential growth phase. The shorter lag phase and more prolonged exponential phase in TMC compared to individual cultures occurred due to using *Pseudomonas* species with *B.* *subtilis*. These results show the positive effect of mixed-culture on the performance of each member.

Evaluation of the kinetic parameters of cell growth indicated that the maximum specific growth rate of *B.* *subtilis* and *P.* *aeruginosa* in TMC were 45.2 and 123.4% higher than in pure cultures (Table 2). Hence, the generation time was lower in TMC and reduced from 7.45 to 5.13 days for *B.* *subtilis* and 14.76 to 6.60 days for *P.* *aeruginosa*. Additionally, the maximum specific growth rate of *P.* *putida* in pure culture and TMC was almost similar (0.107 per day for pure culture and 0.119 per day in TMC), so it exhibited a similar duplication time in both cultures (6.48 and 5.82 days for pure culture and TMC).

**Table 2:** Parameters of cell growth in individual cultures and TMC.

Parameters	µ_max_ (per day)		T_d_ (day)	
	Pyrene	Tetracosane	Pyrene	Tetracosane
*B.* *subtilis*	0.093	0.108	7.45	6.66
*B.* *subtilis* in TMC	0.135	0.114	5.13	6.08
*P.* *putida*	0.107	0.090	6.48	7.70
*P.* *putida* in TMC	0.119	0.112	5.82	6.18
*P.* *aeruginosa*	0.047	0.065	14.76	10.67
*P.* *aeruginosa* in TMC	0.105	0.127	6.60	5.45

Next, three mathematical models (zero, first, and second-order) were tested to describe the kinetic process of pyrene degradation; the experimental data for kinetic model prediction is shown in Fig. 3. In addition, in Table 3, the apparent rate constant (K), half-life period (T_1/2_), and the equation for each kinetic model, along with their corresponding coefficients (R^2^), are presented. The R^2^ is higher for the first-order kinetic of *B.* *subtilis*, *P.* *putida*, and TMC, indicating that the kinetics of pyrene removal follows the first-order model, which implies that the concentration of pyrene decays exponentially with time. However, the kinetic of pyrene removal by *P.* *aeruginosa* followed zero-order kinetics and implied linear reduction of pyrene concentration with time. Therefore, it can be assumed that pyrene removal is a time-dependent process. The removal rate constant (K) is 0.063, 0.068, and 0.123 per day for *B.* *subtilis*, *P.* *putida*, and TMC, respectively. It was also observed that the time needed to remove half of the pyrene (T_1/2_) was smaller in the TMC (5.63 days) than in individual cultures of *B.* *subtilis* (11.01 days), *P.* *putida* (10.19 days), and *P.* *aeruginosa* (41.32 days). These data suggest that pyrene removal is carried out mainly by *B.* *subtilis* and *P.* *putida* because kinetic degradation profiles of TMC and individual cultures of *B.* *subtilis* and *P.* *putida* had more similarities. Hence, it can be concluded that *P.* *aeruginosa* mostly played a supporting role for *B.* *subtilis* and *P.* *putida* by increasing the level of bioavailable pyrene and itself has used more of the intermediate created by *B.* *subtilis* and *P.* *putida*.

**Table 3:** Kinetic parameters of pyrene degradation by individual cultures and TMC.

Parameter	*B.* *subtilis*	*P.* *putida*	*P.* *aeruginosa*	TMC
Zero order equation	Ct = − 22.301t + 533.62	Ct = − 22.743t + 523.94	Ct = − 6.049t + 509.97	Ct = − 31.837t + 505.07
K_0_ (mg/l.day)	22.301	22.743	6.049	31.837
T_1/2_ (day)	11.21	10.99	41.32	7.85
R^2^	0.928	0.917	0.976	0.924
First order equation	LnCt = − 0.063t + 6.333	LnCt = − 0.068t + 6.3281	LnCt = − 0.0132t + 6.2379	LnCt = − 0.123t + 6.3495
K_1_ (per day)	0.063	0.068	0.0132	0.123
T_1/2_ (day)	11.01	10.19	52.51	5.63
R^2^	0.981	0.989	0.903	0.992
Second order equation	1/Ct = 0.0002 + 0.0016	1/Ct = 0.0003t + 0.0015	1/Ct = 0.00003 + 0.0019	1/Ct = 0.0006t + 0.0009
K_2_ (l/mg.day)	0.0002	0.0003	0.00003	0.0006
T_1/2_ (day)	10	6.66	66.66	3.33
R^2^	0.906	0.854	0.875	0.863

**Figure 4:**
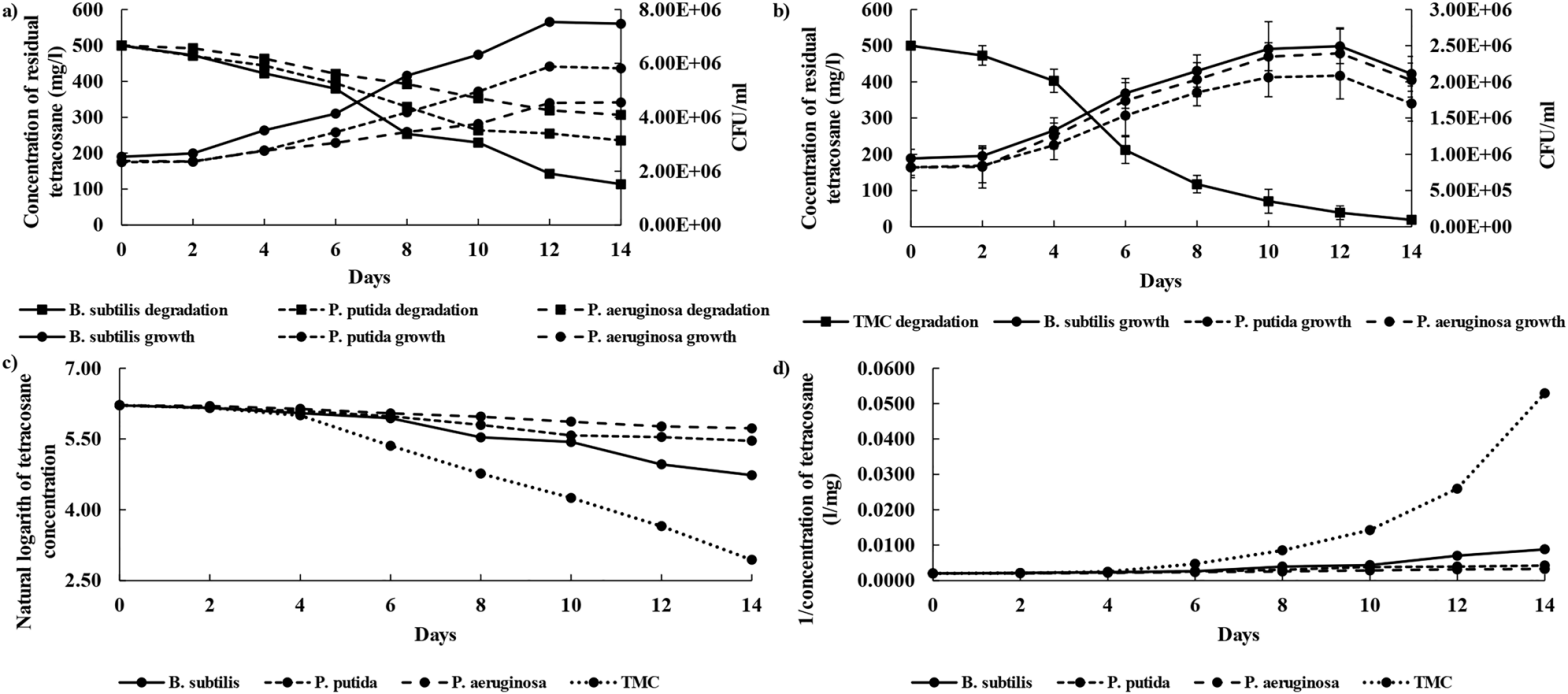
(**a**,**b**) Show the growth rate and degradation yield (zero order kinetic) of tetracosane by pure cultures and TMC. Error bars along each point represent the standard deviation of the mean (n = 3). (**c**,**d**) Show the first order, and second order kinetic modeling of tetracosane degradation by pure cultures and TMC.

Three growth stages for pure cultures and four growth stages for TMC were observed during tetracosane removal (Fig. 4). The growth stages for tetracosane degradation by pure cultures were similar to pyrene degradation by TMC but was no lag phase for *B.* *subtilis*. Finally, the growth stages of tetracosane degradation by TMC included: (1) a 2-day lag phase with a significant amount of degradation; (2) the logarithmic phase with the highest rate of degradation, which lasted for 8 days; (3) a 2-day stationary phase in which degradation rate slightly decreased, and growth rate stopped, and (3) finally, death phase where the biomass reclined greatly but degradation rate was stabilized.

As shown in Table 2, there was no significant difference in the maximum specific growth rate or duplication time of *B.* *subtilis* in TMC compared to its pure culture. However, the maximum specific growth rate of *P.* *putida* and *P.* *aeruginosa* in TMC was approximately 24.5 and 95.4% higher than their pure cultures, and the duplication time was 1.52 and 5.22 days lower. *P.* *aeruginosa* growth in the TMC was significantly higher than in pure culture and even other species, even though it had the slowest growth in individual cultures. This result indicates the *P.* *aeruginosa*’s weakness in the primary attacks on tetracosane molecules and its ability to remove secondary metabolites produced by *B.* *subtilis* and *P.* *putida*. Naturally, the higher growth of *P.* *aeruginosa* will be accompanied by more biosurfactants, which increases the number of bioavailable carbon sources and causes maximum degradation.

The acquired data on tetracosane degradation was adjusted to different kinetic models (Fig. 4), and the respective removal rate constants were obtained. According to the R^2^ (Table 4), it was concluded that the removal of tetracosane fits first-order kinetics, with removal constants of 0.110, 0.60, 0.038, and 0.246 per day, respectively for *B.* *subtilis*, *P.* *putida*, *P.* *aeruginosa*, and TMC. Likewise, it was observed that the time required to remove half of the tetracosane (T_1/2_) is less in TMC (2.82 days) than in the individual culture of *B.* *subtilis* (6.30 days), *P.* *putida* (11.55 days), and *P* *aeruginosa* (18.24 days).

**Table 4:** Kinetic parameters of tetracosane degradation by individual cultures and TMC.

Parameter	*B.* *subtilis*	*P.* *putida*	*P.* *aeruginosa*	TMC
Zero order equation	Ct = − 30.106t + 525.02	Ct = − 21.084t + 509.21	Ct = − 15.347t + 513.44	Ct = − 39.487t + 505.52
K_0_ (mg/l day)	30.106	21.084	15.347	39.487
T_1/2_ (day)	8.30	11.85	16.29	6.33
R^2^	0.907	0.924	0.941	0.894
First order equation	Ct = − 0.110t + 6.4025	Ct = − 0.060t + 6.2728	Ct = − 0.038t + 6.2605	Ct = − 0.246t + 6.6378
K_1_ (per day)	0.110	0.060	0.038	0.246
T_1/2_ (day)	6.30	11.55	18.24	2.82
R^2^	0.973	0.988	0.995	0.980
Second order equation	1/Ct = 0.0005t + 0.0008	1/Ct = 0.0002t + 0.0017	1/Ct = 0.0001t + 0.0019	1/Ct = 0.0031t − 0.0073
K_2_ (l/mg day)	0.0005	0.0002	0.0001	0.0031
T_1/2_ (day)	4	10	20	0.64
R^2^	0.813	0.895	0.931	0.714

## Materials and methods

### Chemicals and culture conditions

Tetracosane, pyrene, and cycloheximide were purchased from Sigma Aldrich (Burlington, Massachusetts, USA). Glucose, R2A, nutrient broth, and salts for BH were obtained from Merck (Darmstadt, Germany). Heavy crude oil was from Gachsaran, Kohgiluyeh and Boyer-Ahmad Province, Iran, and the seawater was sampled from the Persian Gulf with 3.8% w/v salinity. All chemicals were of high quality.

In this study, 14 bacterial strains were isolated from three different regions of Iran (Naft Shahr, Sarkhoon, and Siri). After evaluating the degradation potentials of these bacteria in degrading heavy crude oil, the best strain was selected for further studies. The ability of isolated strains to degrade 1% w/v heavy crude oil was the only criteria for determining the best isolate. Because of the isolation area’s salinity, the sensitivity of the selected strain toward different concentrations of NaCl (0–20% w/v) was investigated. Also, the ability of the selected strain and two *Pseudomonas* species (*P.* *aeruginosa* ATCC 10145 and *P.* *putida* ATCC 12633) in producing and releasing biosurfactant at different carbon sources were evaluated (Supplementary File 1).

Enrichment, growth, and degradation media were BH, a liquid salt-based and carbon-free media, supplemented with glucose, heavy crude oil, pyrene, or tetracosane as carbon sources. Both isolation and store plates were R2A. The growth media for molecular identification was Nutrient Broth. In order to prepare the inoculants, first, a loopful of bacteria was added to 10 ml BH, supplemented with 1% w/v glucose and 0.1% w/v heavy crude oil, then incubated until reaching the optical density of 0.6 at 600 nm (OD600 nm). After that, their biomass was harvested, washed, and suspended in fresh BH. Finally, 1 ml of pre-cultures were inoculated to 9 ml of culture media to form 10% v/v inoculation. The experiments were conducted in triplicate at 28 °C on a rotary shaker (120 rpm). In addition, two sets of flasks, one without inoculation and the other inoculated with inactivated biomass (through sterilization), were used as controls to evaluate losses of carbon source by photolysis, adsorption, and evaporation.

### Degradation of HMW hydrocarbons

In order to evaluate the ability of the selected isolate in removing HMW hydrocarbons, tetracosane and pyrene were used as models for long-chain aliphatics and HMW PAHs. This test examined the efficiency of pyrene and tetracosane degradation at different concentrations (300 up to 1000 mg/l). Two stock solutions of 5000 mg/l pyrene and tetracosane, respectively, in acetone and *n*-hexane, were made to obtain the appropriate concentrations. Then, the calculated volume of stock solutions to obtain the desired final concentration of pyrene and tetracosane was added to 50 ml flasks with 9 ml BH. The flasks were supplemented with 5% w/v NaCl and then incubated overnight on a rotary shaker before inoculation for solvent evaporation. Afterward, one ml of proper pre-culture was added to experimental cultures and incubated for 14 days^48,49^. At the end of the incubation period, the samples were collected. The remaining pyrene and tetracosane were extracted by adding an equal amount of toluene and placed on a rotary shaker for 20 min. This process was repeated three times to remove all the residual hydrocarbons from the environment. Also, before solvent extraction, 100 mg/l of Benzo [a] pyrene and hexacosane were added to the medium as surrogates for pyrene and tetracosane. The extracted hydrocarbons were measured by Gas Chromatography (GC)^50^.

### Gas chromatography

The extracted samples were analyzed by a Gas Chromatograph (Shimadzu GC 8AIT, Kyoto, Japan) equipped with a split-splitless inlet and an FID for separation and determination of pyrene and tetracosane. The inlet temperature was kept at 250 °C. A DB-5 (5 percent phenyl, 95 percent methylpolysiloxane) capillary column (30 m 0.25 mm i.d., df: 0.25 m) for the separations was provided by Shimadzu. Helium (99.999%) was used as the carrier gas at a constant flow rate of 1 ml/min. The oven temperature was programmed as follows: initial temperature of 80 °C (held for 1 min), then increased at a rate of 20 °C/min to 320 °C. The FID temperature was 300 °C. Each sample was replicated three times, and all data were obtained by injecting 1 μl of the extracts^50^.

### Synergistic effect of mixed-culture

*Pseudomonas* *aeruginosa* ATCC 10145 and *P.* *putida* ATCC 12633 were used to study the synergistic effects of bacterial mixed-culture on pyrene and tetracosane degradation. Though the ability of *P.* *aeruginosa* ATCC 10145 in producing biosurfactant and *P.* *putida* ATCC 12633 in degrading HMW hydrocarbons, especially PAHs, have been already established; Their capacity to produce biosurfactant and remove pyrene and tetracosane were evaluated at 5% w/v NaCl (same as the selected strain). The inoculums of two binary mixed-culture consisted of selected strain with *P.* *aeruginosa* (BMC 1) or *P.* *putida* (BMC 2), and ternary mixed-culture (TMC) consists of selected strain with both *Pseudomonas* species were obtained by mixing equal volume of each pre-culture^6^. The prepared inoculums of BMC 1, BMC 2, and TMC were added to two different sets of flasks containing 9 ml BH with 5% w/v NaCl supplemented by 500 mg/l pyrene or tetracosane. After 14 days, the residual pyrene and tetracosane were harvested, as mentioned before, and analyzed by GC.

### The effect of salinity on degradation

Degradation of pyrene and tetracosane by the best strain and the best mixed-culture was measured in seawater and BH at four different salinities (0, 2.5, 5, and 10% w/v NaCl). As mentioned before, the degradation experiments were performed by adding 500 mg/l pyrene or tetracosane to the flasks and performing proper inoculations. The incubation process lasted up to 14 days^23^. The residual hydrocarbons were measured on the 10th, 12th, and 14th days by extracting pyrene and tetracosane with toluene and performing GC analysis.

### Growth rate and kinetics of degradation

The growth rate of three bacterial strain and their kinetics for pyrene and tetracosane degradation were studied as individual and mixed-culture at 5% w/v salinity. The experimental flasks and inoculums of individual cultures and TMC were prepared according to the procedure used in the previous sections. The experiment was carried out for 14 days, and samplings were done every 48 h. The inorganic parts of pyrene and tetracosane flasks were separated using toluene as solvent and sent for GC analysis^6^. The growth of the organic portion was evaluated by the pour plate technique using Nutrient Agar containing 50 mg/l cycloheximide^51^. The kinetic parameters of pyrene and tetracosane degradation were measured using zero-order, first-order, and second-order mathematical models. The kinetic parameters of pyrene and tetracosane degradation were measured using zero-order, first-order, and second-order mathematical models. The curve lines of mathematical models were plotted versus time, and their regressions were considered the acceptance criterion. The following models were applied for the evaluation of pyrene and tetracosane removal kinetics:

1 $${\text{Zero-order\,model:}}\,Ct\, = \, - K_{0} t\, + \,C_{0} \,{\text{and}}\,T_{1/2} \, = \,C_{0} /{2}K_{0},$$

2 $$ {\text{First{-}order}}\,{\text{model:}}\,{\text{ ln}}\,C_{t} \, = \,\, - \,K_{1} t\, + \,{\text{Ln}}C_{0} \,{\text{and}}\,T_{1/2} \, = \,{\text{Ln2}}/K_{1} , $$

3 $$ {\text{Second{-}order model:}}\,{ 1}/Ct\, = \,K_{2} t\, + \,{1}/C_{0} \,{\text{and}}\,T_{1/2} \, = \,{1}/C_{0} K_{2} . $$

In which *Ct* = Concentration of hydrocarbon at time *t*, *K*_0_, *K*_1_, *K*_2_ = Removal rate constant, respectively for zero, first, and second kinetic model, *t* = Time, *C*_0_ = Concentration of hydrocarbon at t_0_, and *T*_1/2_ = Half-life period^48^.

Furthermore, for evaluating the growth profile of each strain in individual cultures or TMC, the upcoming parameters were computed.

4 $$ {\text{Maximum}}\,{\text{specific}}\,{\text{growth}}\,{\text{rate:}}\,\mu_{max} \, = \,\left( {{\text{Ln}}X_{f} {-}{\text{Ln}}X_{i} } \right)/\left( {t_{f} {-}t_{i} } \right), $$

5 $$ {\text{Duplication}}\,{\text{time:}}\,T_{d} \, = \,{\text{Ln2}}/\mu_{max} . $$

In which, *X*_*i*_ is the Concentration of biomass at the beginning of the logarithmic phase, *X*_*f*_ is the Concentration of biomass at the end of the logarithmic phase, *t*_*i*_ is the Time at the beginning of the logarithmic phase, *t*_*f*_ = Time at the end of the exponential phase^48^.

### Statistical analysis

The experimental results were treated statistically by SPSS v24. All the experiments were conducted in triplicate. The Kolmogorov–Smirnov test investigated the normal distribution of obtained results, which showed that all the data were distributed normally. In the tables, data were expressed as mean ± SD. Also, error bars in the figures show standard deviation. Finally, an ANOVA test followed by a post hoc Tukey’s test was used to analyze the differences between means. The significant difference was considered p < 0.05.

## Conclusion

In this paper, *B.* *subtilis* HG 01 was isolated from polluted soils, and its proficiency in the degradation of pyrene and tetracosane was investigated. This strain’s resistance toward salinity (up to 20% w/v NaCl) and its potential to degrade high concentration of pyrene and tetracosane (up to 1000 mg/l) at different salinities (up to 10%) was investigated. The effects of using its mixed culture with *P.* *aeruginosa* and *P.* *putida* and seawater (untreated and treated C/N/P to 100/10/1) on the degradation performance were studied. *B.* *subtilis* HG 01 was able to degrade 55.5 and 77.3% of pyrene and tetracosane at 5% w/v NaCl which signifies the high ability of this bacterium in the degradation process. The cultivation of *B.* *subtilis* HG 01 with *P.* *putida* ATCC 12633, which has the same metabolic pathway, and *P.* *aeruginosa* ATCC 10145, which has a remarkable ability in producing biosurfactant, increased the yield of degradation by about 23%. Also, using supplemented seawater instead of BH media increased the amount of degradation by approximately 17%. The proper function of *B.* *subtilis* HG 01 and its mixed-culture with *Pseudomonas* species in seawater and non-saline environments demonstrated their ability to remove a wide variety of hydrocarbon contaminants in aquatic environments with different salinities. In the end, as a suggestion for future research, it is advised that a comparison be made between the potency of autochthonous microorganisms and allochthonous microorganisms. It can also be beneficial to study the variety and composition of the final metabolites resulting from their degradation and their toxicity.

## Supplementary Information

Supplementary Information 1.

Supplementary Information 2.

Supplementary Information 3.

## Data Availability

The datasets used or analysed during the current study available from the corresponding author on reasonable request. Also, the Raw data of the current are available in a supplementary excel file.
