# Supplementary material for: Biodegradation of high molecular weight hydrocarbons under saline condition by halotolerant Bacillus subtilis and its mixed cultures with Pseudomonas species
Source: Sci Rep. 2022 Aug 2;12:13227. doi: 10.1038/s41598-022-17001-9 (PMC9345985; doi:10.1038/s41598-022-17001-9)
Supplement: Supplementary file 2 — Supplementary Information 2. [file 41598_2022_17001_MOESM2_ESM.docx]

**Enrichment cultures, microbial isolation and degradation of crude oil by isolated strains**

Three soil samples were collected from different areas in Iran (Naft Shahr, Sarkhoon, and Siri), with a long-term history of contamination by various petroleum derivatives. Soil samples were extracted from a depth of 15-20 cm, poured into sterile bags, and transferred to the lab in less than 48 h. In the lab, samples were first dried with hot air, then sieved with a 2 mm strainer, and stored at 4 °. Before the isolation process, a 48 h incubation was conducted for the acclimatization of microbial population to the experimental conditions. For proper enrichment, one gram of soil samples was added to 10 ml distilled water and shook for two h. Then, one ml of resulting solutions was added to 100 ml Bushnell-Haas, supplemented with 1% w/v crude oil (as the only carbon source) and incubated until the accepted absorbance was reached at OD600 nm (OD600 = 0.6). After the incubation period, one ml of 10^-6^ serial dilutions of enrichment media was added to R2A plates, supplemented with 50 mg/l cycloheximide, and incubated for seven days at 28 °C. In the end, 14 bacterial isolates were collected from the soil samples; 9 were gram-positive; 4 were spore-forming. To evaluate the potent of isolated strains in the degradation of petroleum hydrocarbons, they were cultured on a liquid medium, contained heavy crude oil (1% w/v) as the sole carbon source. 50 ml flasks were filled with 9 ml Bushnell-Haas supplemented with 1% w/v heavy crude oil (sole carbon source). Then 10% v/v inoculation was carried out by adding one ml of proper pre-cultures and the flasks were incubated for 14 days. In the end, the residual crude oil was extracted using an equal amount of toluene. The solvent was then evaporated at room temperature, and the amount of remaining crude oil was measured by the gravimetric analysis method. Among 14 isolated bacteria, only six could grow on heavy crude oil. Figure 1 presents the results obtained from the degradation of heavy crude oil. HG 01 exhibited significantly more degradation yield with 59.47%, followed by HG 03 with a 46.27% degradation yield; 13.20% less efficient. The only criterion for determining the best isolate was the effectiveness of the degradation process. Hence, the HG 01 was selected for further studies. This bacterium recovered from an oil-contaminated area in Naft Shahr, Kermanshah province, Iran (34°00'01.9"N, 45°29'33.0"E) and represented as a rod-shaped and gram-positive under microscopic observations and produced a rough, circular colony with slightly yellow color.


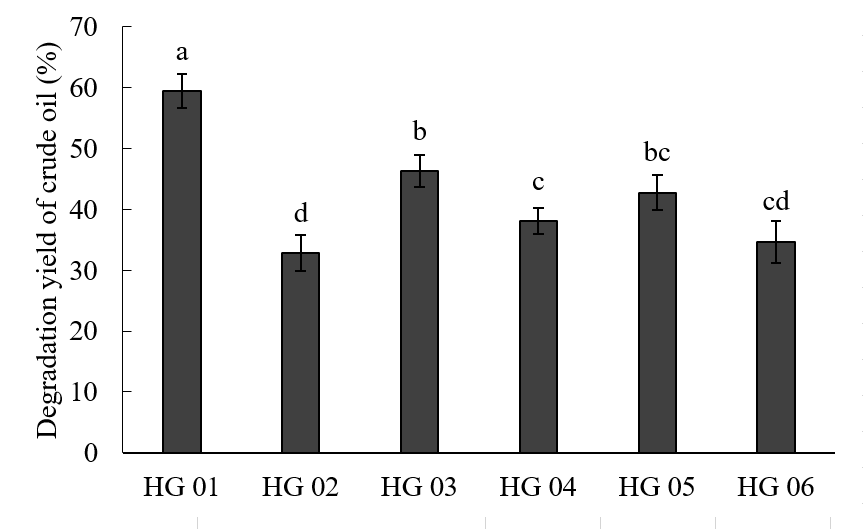


Figure 1: The degradation efficiency of 1% w/v crude oil degradation by isolated bacteria. Data are presented as mean ± S.D. (n = 3). Different alphabets between columns represent significance at p < 0.05 after applying post hoc Tukey's test.

**Molecular Identification of the selected isolate**

To identify HG 01 isolate, a molecular identification process was attained by inoculating a loopful of bacterial cells in a 100 ml flask containing 20 ml Nutrient broth and incubating for 48 h. After that, the bacterial cells were separated from the cultures by centrifugation at 4000 g for 10 min and washed twice with sterilized Ringer's saline. The genomic DNA was extracted using liquid nitrogen and phenol-chloroform methods. Through PCR, and via universal primers 9F (5′-AAGAGTTTGATCATGGCT CAG-3′), and 1541R (5′-AGGAGGTGATCCAACCGCA-3′), approximately 1500 bp of the 16S rRNA gene was amplified. The PCR products were sequenced by the Bioneer Corporation (South Korea) with the same primers. The similarities in the sequences were identified by running a BLAST search in the National Center for Biotechnology Information (NCBI). The results of sequence comparison illustrated 100% similarity to *Bacillus subtilis*. The 16s rRNA sequence of the *B. subtilis* HG 01 was deposited to the genetic sequence database (GenBank) at NCBI with accession number MW548284.

**Growth at different NaCl concentrations**

Different flasks containing 9 ml Bushnell-Haas supplemented with 1% glucose, and various NaCl concentrations (0, 2.5, 5, 7.5, 10, 15, and 20%) were prepared to determine whether *B. subtilis* HG 01 could tolerate the presence of NaCl in liquid culture. Then, one ml of proper pre-culture (to establish 10% v/v inoculation) was added to each flask. The incubation process was carried out over 72 h, and sampling was done at 3, 6, 9, 12, 24, 48, 72 h. Measuring the dry cell weight of biomass was carried out by separating bacterial cells from cultures with centrifugation at 4000 g for 10 minutes. Finally, the recovered biomasses were washed three times with Ringer’s saline, dried at 60 °C (reaching a constant weight), and then weighed. The growth curves of *B. subtilis* HG 01 in different salinities are shown in figure 2. Comparing the diagrams of Figure 2 indicates the better growth of HG 01 in 5% NaCl. However, by increasing the salinity level above 5%, the growth curve endured a declining trend, especially between the range of 10 to 20%, in which a notable growth inhabitation was observed. Despite the severe growth reduction (between 10 to 20% NaCl), HG 01 growth showed an acceptable tolerance against salinity up to 20%.


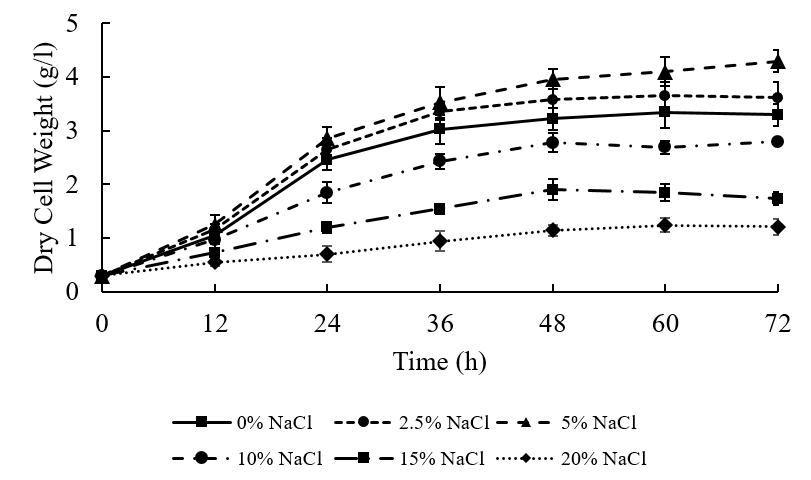


Figure 2: The growth curve of HG 01 strain at different salt concentrations. Data presented as mean ± S.D. (n = 3).

**Production of biosurfactant by *B. subtilis* and *Pseudomonas* species**

To evaluate biosurfactant production under the influence of different carbon sources by *B. subtilis* HG 01 and two *Pseudomonas*species, one ml of pre-cultures was added to 50 ml flasks containing 9 ml BH supplemented with glucose, tetracosane, or pyrene (500 mg/l) and incubated for 14 days. Once the incubation period was over, the ability of bacterial strains to produce biosurfactant was measured. For this purpose, 20 µl crude oil was added to a large plate (20 cm diameter) containing 50 ml distilled water; a 10 µl supernatant of the cultures was added to the surface of the crude oil. After pouring a few drops of the supernatant on the surface, a clear zone would appear if a biosurfactant was present. This process was performed in triplicate, and the diameter of the clear zone was compared with 10 µl distilled water, which acted as the negative control. The ability of *B. subtilis* HG 01, *P. aeruginosa* ATCC 10145, and *P. putida* ATCC 12633 in producing and releasing biosurfactant are shown in Table 1. *B. subtilis* HG 01 could produce biosurfactant on all three carbon sources (Table 1). The oil spreading activity of *B. subtilis* HG 01 supernatants from the pyrene cultures was effectively less than half of the supernatants from glucose and one-third of supernatants from tetracosane. The supernatants of *P. aeruginosa* ATCC 10145 (A well-known biosurfactant producer) had a significant oil spreading on all three carbons sources. Though, its activity decreased about 10 and 32% when the carbon source changed from glucose to tetracosane and pyrene. Also, the supernatant of *P. putida* ATCC 12633 had no statistically significant oil spreading activity.

Table 1: Measurement of oil spreading activity of bacterial cultures supernatant in different carbon sources. Values are mean (n = 3) ± SD. Different alphabets in each column represent significance at p < 0.05 after applying post hoc Tukey's test.

| Strains | Oil spreading test (cm) ± S.D. | | |
| --- | --- | --- | --- |
|  | Glucose | Tetracosane | Pyrene |
| *B. subtilis* HG 01 | 6.48 ± 0.47 a | 9.87 ± 0.67 b | 3.17 ± 0.35 c |
| *P. aeruginosa* ATCC 10145 | 12.06 ± 0.87 d | 10.79 ± 0.23 b | 7.05 ± 0.32 e |
| *P. putida* ATCC 12633 | 1.31 ± 0.18 f | 1.40 ± 0.31 f | 1.23 ± 0.46 f |
| Distilled water | 1.41 ± 0.29 f | 1.19 ± 0.17 f | 1.37 ± 0.24 f |
