# Supplementary material for: Biodegradation of high molecular weight hydrocarbons under saline condition by halotolerant Bacillus subtilis and its mixed cultures with Pseudomonas species
Source: Sci Rep. 2022 Aug 2;12:13227. doi: 10.1038/s41598-022-17001-9 (PMC9345985; doi:10.1038/s41598-022-17001-9)
Supplement: Supplementary file 3 — Supplementary Information 3. [file 41598_2022_17001_MOESM3_ESM.docx]

The ionic composition of Bushnell-Haas broth and Persian Gulf water used in this study

| Persian Gulf Seawater composition (major ion in surface water) | Amount (g L^-1^) | Saline Bushnell-Haas broth medium composition | Amount (g L^-1^) |
| --- | --- | --- | --- |
| Na | 31.36 | Na | 9.27 |
| Cl | 25.73 | Cl | 16.05 |
| Mg | 1.57 | Mg | 0.07 |
| SO_4_ | 2.91 | SO_4_ | 0.13 |
| Ca | 0.64 | Ca | 0.007 |
| K | 0.43 | K | 0.74 |
| HCO_3_ | 0.118 | Fe | 0.017 |
| CO_3_ | 0.016 | NO_3_ | 0.63 |
| Br | 0.072 | PO_4_ | 1.23 |
| N | 0.014 |  |  |
| P | 0.003 |  |  |
| Sr | 0.008 |  |  |
| F | 0.0014 |  |  |
| B(OH)_4_ | 0.0095 |  |  |
| Li | 0.00013 |  |  |
| pH | 7.42 | pH | 7 |
